# The evolution of the lepidosaurian lower temporal bar: new perspectives from the Late Cretaceous of South China

**DOI:** 10.1098/rspb.2009.0030

**Published:** 2009-03-18

**Authors:** Jin-You Mo, Xing Xu, Susan E. Evans

**Affiliations:** 1 Faculty of Earth Sciences, China University of Geosciences, 388 Lumo Road, Wuhan, Hubei 430074, People's Republic of China; 2 Natural History Museum of Guangxi, 1-1 East Renmin Road, Nanning, Guangxi 530012, People's Republic of China; 3 Key Laboratory of Evolutionary Systematics of Vertebrates, Institute of Vertebrate Paleontology and Paleoanthropology, Chinese Academy of Sciences, 142 Xiwai Street, Beijing 100044, People's Republic of China; 4 Research Department of Cell and Developmental Biology, Anatomy Building, University College London, Gower Street, London WC1E 6BT, UK

**Keywords:** lizard, lower temporal bar, *Tianyusaurus*, boreoteiioid, China

## Abstract

Until recently, it was considered axiomatic that the skull of lizards and snakes arose from that of a diapsid ancestor by loss of the lower temporal bar. The presence of the bar in the living New Zealand Tuatara, *Sphenodon*, was thus considered primitive, corroborating its status as a ‘living fossil’. A combination of new fossils and rigorous phylogeny has demonstrated unequivocally that the absence of the bar is the primitive lepidosaurian condition, prompting questions as to its function. Here we describe new material of *Tianyusaurus*, a remarkable lizard from the Late Cretaceous of China that is paradoxical in having a complete lower temporal bar and a fixed quadrate. New material from Jiangxi Province is more complete and less distorted than the original holotype. *Tianyusaurus* is shown to be a member of the Boreoteiioidea, a successful clade of large herbivorous lizards that were dispersed through eastern Asia, Europe and North America in the Late Cretaceous, but disappeared in the end-Cretaceous extinction. A unique combination of characters suggests that *Tianyusaurus* took food items requiring a large gape.

## 1. Introduction

The reptilian group Lepidosauria encompasses Squamata (lizards, snakes and amphisbaenians) and Rhynchocephalia (*Sphenodon* and its fossil relatives). Living members of these two subgroups differ in several respects, but the one most widely cited is the presence in the *Sphenodon* skull of a complete lower temporal bar. For more than a century, the classic view held that the diapsid skull of the ancestral lepidosaur experienced gradual reduction in the lower temporal bar, thereby ‘freeing’ the quadrate (streptostyly) in squamates (e.g. Romer 1956; Robinson 1967). In fact, research over the last 30 years has demonstrated unequivocally that lepidosaurs inherited a skull without a lower temporal bar, although the quadrate was firmly fixed to the skull by the pterygoid and squamosal (e.g. Whiteside 1986; Evans 2003, 2008; Müller 2003). The ancestral lepidosaurian skull architecture was modified differently in each descendant group. The fully diapsid skull of *Sphenodon* is secondary (Whiteside 1986); one or more rhynchocephalian lineages developed a posterior jugal process that ultimately contacted an enlarged anterior quadratojugal process. Squamates, on the other hand, reduced the quadrate/pterygoid and quadrate/squamosal contacts in the evolution of streptostyly. However, so persuasive was the original paradigm with respect to the lower temporal bar that this character in *Sphenodon* is still widely discussed using polarity-related terms such as ‘loss’ or ‘retention’ (e.g. Cleurens *et al*. 1995; Herrel *et al*. 1998, 2007; Schwenk 2000; Metzger 2002; Lü *et al*. 2008). The recent description of a fossil lizard with a complete lower temporal bar, *Tianyusaurus zhengi* (Lü *et al*. 2008), thus seems almost paradoxical, but it highlights the lability of this much-misunderstood character.

*Tianyusaurus* was described on the basis of a single medium-sized specimen, reportedly from the Late Cretaceous of Henan Province, China. Here, we extend the description with three skulls from contemporaneous deposits in Jiangxi Province (Nanxiong Formation, *ca* 66 Myr; see the electronic supplementary material). These skulls are more complete and less distorted than the holotype, and permit this remarkable lizard to be placed more fully into phylogenetic and functional context. Two skulls are significantly larger than the holotype and one is smaller.

## 2. Description and comparative anatomy

Squamata (Oppel 1811)

Boreoteiioidea (Nydam *et al*. 2007)

*T. zhengi* (Lü *et al*. 2008)

*Type specimen*. Shandong Tianyu Natural Museum-05-f702 (Pingyian, Shandong Province, China), a skull and mandible in articulation with the first eight cervical vertebrae and pectoral girdle from the Late Cretaceous Qiupa Formation, Henan Province, China (Lü *et al*. 2008).

*Referred specimens*. Guangxi Natural History Museum, Zoology Collection, Guangxi Province, China, NHMG 8502 (SL=87.4 mm), NHMG 9317 (SL=79.5 mm) and NHMG 9316 (SL=56 mm), from the Late Cretaceous Nanxiong Formation, Ganzhou, Jiangxi Province, China.

*Diagnosis* (emended and extended from Lü *et al*. 2008). A large squamate, with a maximum midline skull length over 87 mm, which resembles other boreoteiioid squamates (*sensu* Nydam *et al*. 2007) in having vomers and pterygoids meeting in the palatal midline; suborbital fenestra reduced by expansion of the ectopterygoid; no palatal dentition; well-developed pterygoid lappet on quadrate; large medially directed adductor fossa; deep vertical pterygoid flanges; clavicle greatly expanded with fenestrate medial end. Resembles many Asian boreoteiioids in having post-caniniform teeth labiolingually compressed and multicuspid; resembles *Gilmoreteius*, *Tuberocephalosaurus* and *Aprisaurus* (Alifanov 2000) in having enlarged maxillary caniniform teeth. Differs from other boreoteiioids and all other squamates in the following combination of derived characters: long posterior process of jugal forming complete lower temporal bar; tympanic crest of quadrate rugose with expanded anteroventral margin; deep fixed quadrate–pterygoid overlap; squamosal and supratemporal fused or partially fused and firmly attached to quadrate dorsal condyle; premaxilla excluded from ventral narial margin by expanded anterior maxillary process; upper temporal fenestra small; lower temporal fenestra and post-temporal fenestra large; lower jaw relatively shallow so that the deep vertical pterygoid flange extends below the lower margin of the closed mandible.

## 3. Morphology

Lü *et al*. (2008) described the main features of the skull of *Tianyusaurus*. Here, we focus on details not reported, or misreported, in the original description and those features of the skull that, together, seem to contribute to a functional explanation of the unusual morphology. More detailed descriptions and illustrations of individual Jiangxi specimens can be found in the electronic supplementary material.

The skull of *Tianyusaurus* is fully diapsid with small dorsally placed upper temporal fenestrae and large post-temporal and lower temporal fenestrae (figures 1 and 2). The choana is not subdivided and the suborbital fenestrae of mature specimens are reduced in width by expansion of the ectopterygoid and pterygoid. Lü *et al*. (2008) figured the holotype skull as anteriorly deep and described the post-temporal fenestrae as dorsoventrally shallow and the jugal bar as bulging. However, that skull has been distorted by lateral compression and breakage. The Jiangxi *Tianyusaurus* specimens have shallower skulls, not unlike those of *Sphenodon* in general shape, and, judging from the breakage patterns, they are closer to the original shape.

**Figure 1:**
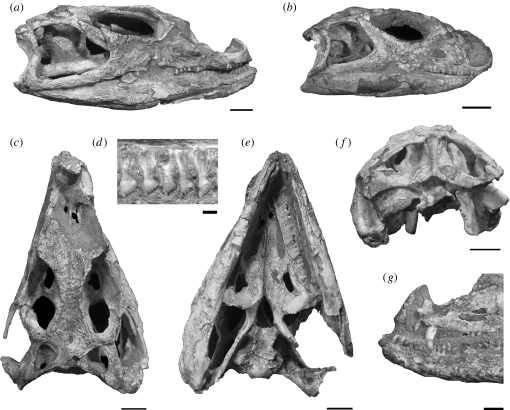
*Tianyusaurus* sp., Jiangxi Province. (*a*) NHMG 8502, right lateral view; (*b*) NHMG 9316, right lateral view; (*c*) NHMG 8502, dorsal view; (*d*) NHMG 9316, detail of maxillary dentition; (*e*) NHMG 8502, palatal view; (*f*) NHMG 9317, occipital view; (*g*) NHMG 8502, detail of anterior skull with caniniform. All scale bars are 10 mm, except (*e*) 5 mm.

**Figure 2:**
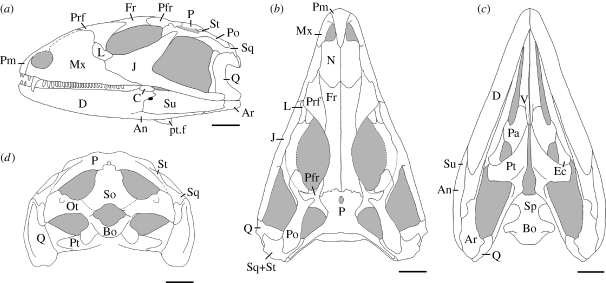
*Tianyusaurus* sp.: reconstruction of the skull in (*a*) left lateral, (*b*) dorsal, (*c*) occipital and (*d*) palatal views, based mainly on NHMG 8502 and NHMG 9317. Scale bars, 10 mm. A, angular; Ar, articular; Bo, basioccipital; D, dentary; Ec, ectopterygoid; Fr, frontal; J, jugal; L, lacrimal; Mx, maxilla; N, nasal; Ot, oto-occipital; P, parietal; Pa, palatine; Pm, premaxilla; Pfr, postfrontal; Po, postorbital; Prf, prefrontal; Pt, pterygoid; pt.f, pterygoid flange; Q, quadrate; So, supraoccipital; Sp, Splenial; Sq, squamosal; St, supratemporal; Su, surangular; V, vomer.

The skull is robust with strong sutures. Anteriorly, the maxilla forms nearly three-quarters of the narial margin. Its long, dorsally concave premaxillary process expands anteriorly to brace the premaxilla, meeting the contralateral maxilla in the posterior midline. The facial process has a procumbent anterodorsal edge overhanging the naris. In NHMG 9317 and NHMG 9316, the post-orbital is separated from the parietal by a slender supratemporal, but in NHMG 8502 the post-orbital expands over the supratemporal to meet the parietal. The quadrate is large with a rugose tympanic crest (NHMG 9317, figure 1) meeting the jugal ventrally and squamosal dorsally in a fixed articulation. The thickened crest may have given origin to posterior fibres of the adductor mandibulae externus superficialis (MAMES). A large medial quadrate wing has an overlapping suture with the pterygoid. The palate bears no teeth (figure 1). The vomer and palatine have a main transverse suture, but (*contra* Lü *et al*. 2008) the latter are separated in the midline by a narrow vomerine–pterygoid contact. The palatine meets the maxilla anteriorly and an enlarged ectopterygoid posteriorly, excluding the maxilla from the narrow suborbital fenestra in palatal view. The pterygoids and ectopterygoids together contribute to long narrow ventrolateral pterygoid flanges that extend below the inferior margin of the closed lower jaw (see fig. S6*c* in the electronic supplementary material). In addition, a sharp pterygoid crest crosses the posteroventral surface of the pterygoid plate, in an anteromedial to posterolateral orientation (see fig. S8 in the electronic supplementary material). By comparison with extant taxa, this crest marked the anterior limit of origin of the aponeurosis to which superficial fibres of the pterygoideus muscle were attached. However, a groove running posterior and parallel to this crest suggests fibres, perhaps of the deep pterygoideus, passed around the pterygoid neck from a dorsomedial attachment. This would increase their functional length.

The sphenoid and basioccipital are fused in mature specimens, but the latter is the shorter element in NHMG 9316. It has a large laterally placed basal tubera. In the sphenoid, a short vidian canal perforates the base of each basipterygoid process from a large posterior foramen. The supraoccipital has a steep posterodorsal margin with a median column that abuts the parietal (figures 1 *f* and 2 *b*; electronic supplementary material). In the oto-occipital, the metotic fissure is divided, as in all squamates, into a small vagus foramen and an elongate lateral opening of the recessus scalae tympani. The latter is framed by a low crista tuberalis and by a narrow occipital recess in the basioccipital. The long, posterolaterally directed paroccipital processes have expanded distal tips meeting the suspensorium. One or two hypoglossal foramina perforate the exoccipital. The prootic has a well-developed crista prootica.

The dentary is supported ventrally by a large angular and braced medially by a plate-like splenial extending from the symphysis to the coronoid. The latter has a low coronoid process, a small lateral lappet and a posterior crest. The surangular is shallow anteriorly, but deepens posteriorly. Its most notable feature is a strong lateral crest that divides the bone into a shallow ventrolateral surface that meets the angular and a broad dorsal shelf or platform that is angled from dorsomedial to ventrolateral. The platform is most marked in mature specimens and bears a posteriorly deepening concavity such that the posterodorsal surface is almost horizontal (see figures and cross section in fig. S4 in the electronic supplementary material). Its lateral edge is in line with the lower temporal bar (rather than bowed) and marks the ventrolateral edge of the adductor chamber (NHMG 9316). By comparison with modern lizards, this broad surface gave insertion to fibres of the 1b portion of the MAMES and perhaps part of the medialis component (MAMEM). The inner edge of the platform forms the dorsal rim of a large medially directed adductor fossa (MAME posterior), the ventral margin of which is formed by the long pre-articular. A shallow anteromedial to posterolateral groove curves onto the lateral jaw surface and probably marks the course of the main (superficial) part of the pterygoideus muscle, whereas a concavity along the ventromedial margin of the articular, associated with a short medially directed crest, probably accommodated deep pterygoideus fibres (Throckmorton 1976, 1978). The articular is short, with a transverse joint surface that is inclined at an anterodorsal to posteroventral angle to the long axis of the jaw. The retroarticular process is reduced to a tubercle in adult specimens.

The premaxilla bears 6–7 small teeth, the dentary approximately 33 and the maxilla approximately 24. The smaller anterior teeth and the large maxillary caniniforms are monocuspid. The post-caniniforms are deeply pleurodont, labiolingually compressed and have expanded multicuspid crowns. The maxillary teeth are most clearly preserved in NHMG 9316 (figure 1 *d*) and are asymmetrical, with a large posterior cusp and a series of smaller cusps (usually four) along the oblique anterior edge. The cuspidation on the dentary teeth is more symmetrical.

The smallest skull, NHMG 9316, shows differences that are probably ontogenetic: retention of braincase sutures; weaker development of crests and rugosities; more rounded upper temporal fenestrae; a larger suborbital fenestra; no maxillary caniniforms; a less inclined quadrate; a weaker jugal–quadrate contact; and a small remnant of the retroarticular process. Lesser differences exist between NHMG 8502 and NHMG 9317, and between these large adults and the Henan holotype, but, allowing for preservation and taphonomy, these are most parsimoniously interpreted as individual variation.

## 4. Phylogenetic analysis

*Tianyusaurus* is a lepidosaur (quadrate with conch; large maxillary facial process; pleurodonty), and its suspensorium (reduced squamosal, supratemporal expanded paroccipital process), braincase (vidian canal, divided metotic fissure) and teeth (deeply pleurodont) show that it is a squamate, as Lü *et al*. (2008) concluded. These authors did not run a cladistic analysis but noted that *Tianyusaurus* might be related to teiioids (figure 3).

**Figure 3:**
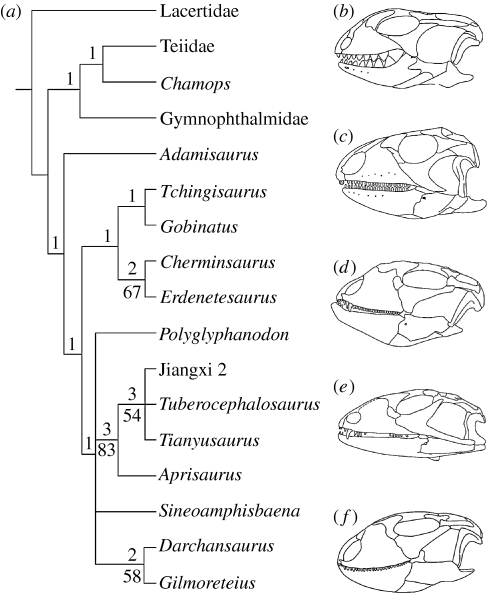
Phylogenetic position of *Tianyusaurus* sp. (*a*) Cladogram showing hypothesis of relationship for *Tianyusaurus* based on a branch and bound analysis of 19 squamate taxa. Small numbers above nodes are decay indices; below nodes are bootstrap values (see the text and the electronic supplementary material for further details). (*b–f*) Left lateral views of Late Cretaceous boreoteiioid skulls (not to scale): (*b*) *Adamisaurus* (Mongolia), (*c*) *Polyglyphanodon* (USA), (*d*) *Tuberocephalosaurus* (Mongolia), (*e*) *Tianyusaurus* (China) and (*f*) *Gilmoreteius* (Mongolia; *Macrocephalosaurus*). Skulls (*b*), (*c*) and (*f*) are redrawn from Estes (1983) and (*d*) from Alifanov (2000).

We coded *Tianyusaurus* into the data matrix of Conrad (2008) and ran a preliminary heuristic analysis (PAUP* v. 4.0b10; Swofford 2002) with 148 exemplars (364 characters) representing all major squamate lineages and several fossil taxa. All but 20 characters were run unordered (as in Conrad 2008), multistate taxa were treated as uncertainty, and Kuehneosauridae, *Marmoretta* and Rhynchocephalia were outgroups. The strict consensus of 1501 equally parsimonious trees (*L*=2969, CI=0.169, RC=0.111) unequivocally placed *Tianyusaurus* with Boreoteiioidea (*sensu* Nydam *et al*. 2007; Polyglyphanodontidae of Conrad 2008). A branch and bound analysis was then performed on a subset of 19 fossil and living teiioid and boreoteiioid taxa, with Lacertidae as the designated outgroup. It yielded four maximum parsimony trees (*L*=354, CI=0.588, RC=0.311) in which the *Tianyusaurus* was consistently placed with the Mongolian genera *Tuberocephalosaurus* and *Aprisaurus*, and a second, as-yet unnamed Jiangxi lizard (Jiangxi 2 in figure 3 *a*), in a subgroup of boreoteiioids. Boreoteiioidea emerged as the monophyletic sister group of Teiidae+Gymnophthalmidae+*Chamops* (Late Cretaceous, North America), a result closer to that of Nydam *et al*. (2007) than to Conrad (2008). However, bootstrap and decay analyses provided only weak support for all clades except Gymnophthalmidae and that comprising the tuberocephalosaurus (including *Tianyusaurus* and the second Jiangxi taxon), and a larger analysis of boreoteiioid relationships is needed.

## 5. Discussion

### (a) Phylogenetic relationships

*Tianyusaurus* is nested within Boreoteiioidea (Nydam *et al*. 2007), a successful group of mainly herbivorous Late Cretaceous lizards encompassing the Euramerican transverse-toothed polyglyphanodontines (e.g. *Polyglyphanodon*; Gilmore 1942) and a more diverse East Asian assemblage including *Adamisaurus* (Sulimski 1972), gilmoreteiids (macrocephalosaurines; Langer 1998), and the smaller ‘mongolochamopines’ (Alifanov 2000), the monophyly of which is untested. Boreoteiioids are first recorded in the Neocomian of Japan (Evans & Manabe 2008) and their roots may be Asian. They represent the largest known radiation of herbivorous lizards, but also the only Late Cretaceous terrestrial lizard clade that did not survive the K-T extinction.

### (b) Functional morphology

*Tianyusaurus* shares many traits with other large Asian boreoteiioids (vomer–pterygoid contact, large jugal, reduced suborbital fenestra, posteriorly extended post-orbital, long paired frontals, short parietal, probable absence of meso- and metakinesis), but it has a shallower skull and lower jaw, longer maxilla, less massive jugal, more extensive quadrate–pterygoid overlap and no retroarticular process. With the exception of *Polyglyphanodon* (Gilmore 1942), no other boreoteiioid is known to elongate the posterior jugal ramus, and none appears to have completely immobilized the quadrate. Many living herbivorous lizards (and presumably boreoteiioids) stabilize their quadrates during powerful static biting using a combination of temporal ligaments and muscle action (Cleurens *et al*. 1995; Herrel *et al*. 1998). This is possible because the lizard quadrate generally rotates posteriorly during jaw closure (e.g. Cleurens *et al*. 1995; Herrel *et al*. 1998; Schwenk 2000; Moazen *et al*. 2008), putting the temporal ligaments into tension (Herrel *et al*. 1998) as the animal bites. By contrast, in crocodiles (Cleurens *et al*. 1995) and *Sphenodon* (Jones 2008; Schaerlaeken *et al*. 2008), forces on the quadrate tend to be directed anteriorly during biting, albeit for different reasons in each, putting the lower skull margin into compression. Under those conditions, a bony lower temporal bar rather than a ligament provides a better mechanical solution (Cleurens *et al*. 1995). Lü *et al*. (2008) concluded that the same argument must apply to *Tianyusaurus*. We agree that this may be the case, but it raises the question as to why the skull of *Tianyusaurus* was loaded so differently from that of its immediate relatives.

A tendency towards predominantly anterior rather than posterior rotation of the quadrate during biting could result from one of several changes in morphology and/or feeding strategy: (i) a cropping action involving strong backward head movements needed to pull plant material from its source (and thus a strong anteriorly directed food resistance force), (ii) an increase in bite force concomitant with an anteriorly directed food reaction force (as in examples cited by Cleurens *et al*. 1995 and Herrel *et al*. 1998), (iii) the development of pro-oral shear (as in *Sphenodon*: Gorniak *et al*. 1982; Jones 2008), or (iv) the development of a powerful pterygoideus muscle (Cleurens *et al*. 1995). A cropping action cannot be ruled out for *Tianyusaurus*, but the small size of the anterior teeth and the absence of any strong occipital crests suggestive of powerful neck muscles render this unlikely as a primary explanation. The large lower temporal and subtemporal fenestrae, expanded post-temporal fenestrae, large medially directed mandibular adductor fossa and robust anterior quadrate rim indicate that *Tianyusaurus* had well-developed external adductor muscles, but probably no more so than its boreoteiioid relatives (with deeper, apparently more powerful jaws). The broad subhorizontal surangular platform in *Tianyusaurus* suggests the superficial (1b) part of the external adductor was thick but, *contra* Lü *et al*. (2008), the lower temporal bar ran in line with the edge of the surangular platform rather than bowing laterally. This would have limited extension of MAMES onto the lateral surface of the mandible (Rieppel & Gronowski 1981), restricting fibre lengths and, potentially, bite force (Schaerlaeken *et al*. 2008), although the latter needs confirmation. The only jaw muscle with an alignment capable of exerting powerful anteriorly directed forces on the quadrate–mandible during jaw closure is the pterygoideus (Throckmorton 1978). In *Sphenodon*, this powerful muscle is primarily responsible for anterior (pro-oral) translation of the mandible, as permitted by an elongated joint surface on the articular (Robinson 1976). The *de novo* development of a lower temporal bar in *Sphenodon* is generally linked to the need to stabilize the quadrate during pro-oral shearing (Gorniak *et al*. 1982; Jones 2008; Schaerlaeken *et al*. 2008). A similar explanation might seem appropriate for *Tianyusaurus*, except that its short, posteroventrally inclined quadrate–articular joint was incapable of anteroposterior sliding. Nonetheless, pterygoideus can contribute to both jaw opening and jaw closure (Frazzetta 1962; Throckmorton 1978; Gorniak *et al*. 1982; Sinclair & Alexander 1987; Schwenk 2000; Reilly *et al*. 2001; McBrayer & White 2002), and the Jiangxi *Tianyusaurus* skulls show clear traces of deep and superficial pterygoideus origin and insertion (anterior pterygoid crest, medial ridge and recess on articular, lateral surangular crest and strong pterygoid flange).

Other unusual features of the *Tianyusaurus* skull morphology may be relevant. All boreoteiioids have strong pterygoid flanges, but in *Tianyusaurus* these flanges are narrow and extend below the level of the jaw margin when the mouth is closed. This is an unusual trait in lizards, but it does occur in chameleons (S. E. Evans 2008, personal observations) that have a large gape (Herrel *et al*. 2001), the flange guiding and stabilizing jaw movements, resisting lateral displacement. Chameleons also reduce the retroarticular process. In *Tianyusaurus*, the reduced retroarticular process (Frazzetta 1962), together with the posteroventrally angled jaw joint and fixed quadrate (Throckmorton 1976), would facilitate a wide gape. In an akinetic non-propalinal skull (e.g. *Caiman*: Sinclair & Alexander 1987), pterygoideus operates at maximum mechanical advantage when the mouth is wide open. Biting with an open mouth and a fixed quadrate, with pterygoideus activated, would load the lower margin of the skull in compression. A framework of lateral bracing bars would also stabilize the skull against torsional forces. Instead of relying on a deep robust skull and jaw to crop tough plant material (as in other boreoteiioids), *Tianyusaurus* may have taken food items that required a large gape and an ability to exert a penetrating force at the early stages of the bite (e.g. turgid fleshy fruits). This would also be consistent with the differentiation of the upper and lower teeth. The asymmetrical upper teeth seem ideally shaped to perforate (apical cusp) and then cut (oblique cusped blade) as the jaws close, while the lower teeth are better shaped to hold food in the mouth. The large caniniforms, also present in other large boreoteiioids, are most likely to be associated with territorial behaviour, but some living lizards use such teeth to grip large food items (Torres-Carvajal 2007). According to Tinsley (2004), the Late Cretaceous saw an increase in size of angiosperm fruits in addition to the fleshy fruits of ginkgos, and some conifers, cycads and seed ferns. This may be relevant.

Clearly, *Tianyusaurus* had a feeding strategy that set it apart from its boreoteiioid relatives. The hypothesis of the skull function set out above needs to be tested (e.g. with computer modelling), with a broader survey of boreoteiioid skull and dental morphology and phylogeny. Nonetheless, the discovery of a squamate with a complete lower temporal bar emphasizes the lability of functional characters and the need to understand the biomechanical and developmental constraints that operate on skulls as they evolve.
